# Clinical use of ^11^C-methionine and ^18^F-FDG-PET for germinoma in central nervous system

**DOI:** 10.1007/s12149-013-0787-4

**Published:** 2013-11-24

**Authors:** Yoshiyuki Okochi, Takashi Nihashi, Masazumi Fujii, Katsuhiko Kato, Yumiko Okada, Yoshio Ando, Satoshi Maesawa, Shigenori Takebayashi, Toshihiko Wakabayashi, Shinji Naganawa

**Affiliations:** 1 Department of Radiology, Nagoya University Graduate School of Medicine, 65 Tsurumai-cho, Shouwa-ku, Nagoya, 466-8550 Japan; 2 Department of Neurosurgery, Nagoya University Graduate School of Medicine, Nagoya, Japan; 3 Department of Radiological and Medical Laboratory Sciences, Nagoya University Graduate School of Medicine, Nagoya, Japan; 4 Departments of Radiology, Japanese Red Cross Nagoya Daiichi Hospital, Nagoya, Japan; 5 Department of Neurosurgery, Sakura General Hospital, Niwa, Aichi Japan; 6 Departments of Neurosurgery, Nagoya Central Hospital, Nagoya, Japan

**Keywords:** Germinoma, Positron emission tomography, ^11^C-methionine, ^18^F-FDG

## Abstract

**Objective:**

The purpose of this study was to examine the ^11^C-methionine (MET) and ^18^F-fluorodeoxyglucose (FDG) positron emission tomography (PET) findings of central nervous system (CNS) germinoma and the diagnostic utility of these findings.

**Methods:**

We retrospectively evaluated the cases of 10 patients who were diagnosed with CNS germinoma according to their histopathological or clinical findings. All the patients underwent pretreatment MET and/or FDG-PET scans, and the resultant images were assessed qualitatively and quantitatively. In the qualitative assessments, we used 3- and 5-grade visual scoring systems for the MET- and FDG-PET images, respectively. In the quantitative assessments, the maximal standardized uptake value (SUV_max_) and the ratio of the SUV_max_ of the tumor (*T*) divided by the mean SUV for the normal white or gray matter [*T*/*N* (WM), *T*/*N* (GM)], was calculated.

**Results:**

The mean and SD values of SUV_max_, *T*/*N* (WM), and *T*/*N* (GM) were 1.9 ± 1.4, 2.5 ± 1.3, and 1.7 ± 0.9 on MET-PET and 5.8 ± 2.2, 1.6 ± 0.5, and 0.8 ± 0.2 on FDG-PET, respectively. On MET-PET, only one lesion was not detected. On the other hand, on FDG-PET all of the lesions exhibited uptake values that were intermediate between those of the normal white matter and gray matter.

**Conclusion:**

In terms of its tumor-contouring ability, MET is a good tracer for diagnosing CNS germinomas; therefore, MET-PET is considered to be useful for planning biopsies or surgery. Although FDG-PET is capable of detecting CNS germinomas, it produced insufficient image contrast in the present study. Further studies are needed before FDG-PET can be used in clinical examinations of CNS germinoma.

## Introduction

Germinoma, teratoma, choriocarcinoma, embryonal cell carcinoma, yolk sac tumor, and mixed tumors are all types of germ cell tumor (GCT). In the USA, GCT accounts for 0.5 % of all primary brain and central nervous system (CNS) tumors [1]. However, GCTs are more common in East Asia than in Western countries. For example, GCT accounts for 3.1 % of all primary brain tumors in Japan [2].

The prognosis of primary GCT varies depending on the histology and size of the tumor as well as its extent at the initial diagnosis [3]. In general, intracranial GCTs are classified into three categories, i.e., those with good, intermediate, and poor prognoses [4]. Malignant GCT, such as embryonal carcinoma, yolk sac tumors, immature teratomas, teratomas exhibiting malignant transformation, and mixed tumors, generally exhibit poor prognoses [5–7]. On the other hand, CNS germinoma patients tend to display a good prognosis. Germinoma with syncytiotrophoblastic giant cells (STGC) is a subtype of CNS germinoma that is considered to have an intermediate prognosis. STGC produce β-HCG; therefore, the detection of β-HCG in serum or cerebrospinal fluid (CSF) is an indicator of germinoma with STGC [8]. CNS germinomas are generally sensitive to radiotherapy and chemotherapy, and their 10-year survival rate is approximately 90 %; i.e., most of them are curable [9, 10].

CNS germinomas tend to develop in the midline structures of the brain, such as the pineal gland or the suprasellar region, and often occur as multifocal or disseminated lesions. In rare cases, CNS germinomas develop at ectopic sites such as the basal ganglia, thalamus, or internal capsule [11–16]. If germinoma is suspected, magnetic resonance imaging (MRI) is an essential diagnostic neuroimaging modality, and biopsy is recommended for achieving a final diagnosis [3, 10]. However, biopsies are sometimes omitted in cases in which the lesion exhibits a characteristic location and tumor markers such as β-HCG and alpha-fetoprotein (AFP) show appropriate expression patterns (tests for β-HCG are negative or slightly positive and those for AFP are negative) [17]. In germinoma patients in whom a biopsy would be a high-risk procedure, neoadjuvant therapy with very low-dose irradiation or chemotherapy might be helpful for confirming the presumed diagnosis. [10]. In addition, non-invasive neuroimaging can also play an important role in pretreatment examinations.

Typical CNS tumors are easy to diagnose by conventional MRI; however, in some cases diagnosis is delayed due to the slow progression of the lesion or the tumor being located in the basal ganglia, in which it is difficult to detect tumors because lesions in this region only exhibit subtle signal changes on MRI during their early stages. On the other hand, many kinds of tumor can occur in the suprasellar region, e.g., craniopharyngioma, pituitary adenoma, glioma, hypophysitis, sarcoidosis, and germ cell tumor, all of which exhibit similar shapes and signal changes on MRI; therefore, the differential diagnosis of suprasellar tumors is often difficult.

The usefulness of ^11^C-methionine (MET)-positron emission tomography (PET) for tumor contouring and treatment planning has been extensively investigated in patients with brain tumors [18–20], and ^18^F-fluorodeoxyglucose (FDG)-PET is widely used for differential diagnosis, staging, histological grading, and prognostic evaluations in many kinds of tumor [21]. In typical cases of germinoma, PET imaging is considered to be of limited use; however, some difficult cases cannot be diagnosed by conventional MRI. Previous reports have indicated that MET-PET might facilitate the early diagnosis of CNS germinoma; however, these reports only assessed basal ganglia germinomas [9, 11, 12, 15, 16]. On the other hand, there have not been any systematic reports about the utility of FDG-PET in cases of CNS germinoma. Therefore, we elucidated the characteristic MET- and FDG-PET findings of germinomas that develop in the basal ganglia or the suprasellar or pineal region; i.e., the regions of the CNS that are most commonly affected by germinoma.

Specifically, we assessed the uptake of MET and FDG by CNS germinomas in the pineal gland, the suprasellar region, and the basal ganglia.

## Materials and methods

### Patients

Between November 2007 and March 2011, 10 CNS germinomas were initially diagnosed at our institution on the basis of their pathological findings or clinical features. All of these tumors were assessed by MET-PET and/or FDG-PET.

Pathological diagnoses (pure germinoma in all cases) were obtained from biopsy specimens in 5 cases. In the other 5 cases, we diagnosed the germinomas according to their clinical features. Based on the locations of the tumors; the patients’ age, sex, and tumor marker levels; and the fact that none of the patients exhibited positivity for AFP, which were suggestive of immature teratoma or mixed germ cell tumor, we administered a single course of chemotherapy and then assessed the treatment response. On the basis of the aforementioned clinical findings, the latter 5 tumors were diagnosed as germinomas.

Our study was approved by the Committee for Ethics of Nagoya University School of Medicine.

### PET procedure

Positron emission tomography images were obtained using a Headtome-V PET camera (Shimadzu, Kyoto, Japan) with a spatial resolution of 4.5 mm (axial) and a full width at half maximum of 3.9 mm (transaxial) in the center of the field of view. The corrected data were reconstructed into 31 transaxial planes with a slice thickness of 6.25 mm and a 256 × 256 pixel image matrix. The effects of soft tissue attenuation on the scans were corrected using transmission scans that utilized a rotating 68 Ge/68 Ga line source. A 5-min transmission scan was performed before the PET tracer injection, and 60-min (5 min × 12 frames) and 40-min (2 min × 20 frames) emission scans were started after the intravenous injection of FDG and MET, respectively. We analyzed the tracer uptake of the tumors by qualitative and quantitative methods. We used 11–20 frames to analyze tracer uptake during the MET-PET and 9–12 frames to examine it during the FDG-PET.

All of the subjects fasted for at least 6 h prior to the FDG-PET. In addition, they were asked to close their eyes and to remain silent throughout the scanning procedure, which was performed whilst they were in the supine position. The dose of PET tracer was 11.1 and 3.7 MBq/Kg for the MET- and FDG-PET scans, respectively. All emission scans were performed in 2D acquisition mode, and attenuation-corrected images were reconstructed with filtered back projection.

The maximum period between the MET- and FDG-PET scans was 1 week, and no interventions were performed between the two PET scans.

### Analysis of the PET images

The region of interest (ROI) was drawn manually so that it included the part of the germinoma that exhibited the highest tracer uptake as well as normal brain tissue as a control. We visually identified regions of normal gray and white matter in the contralateral supratentorial hemisphere (Y.O., T.N.). If the tumor borders were unclear, we carefully chose the ROI using MRI, including gadolinium (Gd)-enhanced T1- or T2-weighted imaging. In cases in which the tumor had a distorted shape and/or was small, there was a risk that the tumor’s tracer uptake would be underestimated due to the partial volume effect [22]. Therefore, we carefully measured tracer uptake after referring to PET and MRI images to accurately determine the peak uptake value.

In the qualitative assessments, tracer uptake was evaluated using visual scoring systems. For MET-PET, the degree of MET uptake was scored as follows: grade 1 (negative), grade 2 (minimally positive), or grade 3 (positive); negative indicated that the MET uptake was markedly lower than the background value, and positive indicated that the MET uptake was markedly higher than the background value. Minimally positive referred to uptake that was neither positive nor negative. For FDG-PET, the degree of FDG uptake by the brain lesions was scored as follows: grade 1 (<normal white matter), grade 2 (=normal white matter), grade 3 (between those of the normal white matter and normal gray matter), grade 4 (=normal gray matter), or grade 5 (>normal gray matter).

In the quantitative assessments, tracer uptake by the tumor was evaluated using the maximal standardized uptake value (SUV_max_), the tumor-to-normal gray matter (*T*/*N* (GM)) ratio, and the tumor-to-normal white matter (*T*/*N* (WM)) ratio. The *T*/*N* ratio was defined as the ratio of the SUV_max_ of the tumor (*T*) ROI divided by the mean SUV (SUV_mean_) for the normal gray matter or white matter (contralateral frontal cortex) (*N*). The maximum SUV and mean SUV were calculated as follows: SUV_max_ = maximum RI count (Bq/ml)/[MET dose (Bq)/body weight (g)]; SUV_mean_ = mean RI count (Bq/ml)/[MET dose (Bq)/body weight (g)]. We measured the uptake values of all of the germinoma lesions.

The size of the normal brain tissue ROI differed from case to case. During the determination of the ROI for the GM, we carefully traced a gyrus reference from anatomical MRI and calculated the mean SUV. On the other hand, for the WM we first identified the ventricle and a GM reference on MRI and then drew the WM ROI so that it did not include the GM or ventricle and was as large as possible.

Comparisons between the tracer uptake values for all patients and those for the patients with verified pathological diagnoses (No. 2, 4, 5, 9, and 10) were also made.

### Statistical analysis

For each tumor type, the mean and standard deviation (SD) of the visual scores and the SUV values were calculated. These values were compared using an unpaired *t* test. *P* values of <0.05 were considered significant. All analyses were performed with SPSS 17.0.

## Results

### Patients

Ten germinoma patients (8 males and 2 females; mean age 13.4 ± 7.1) were involved in the present study. Eight of the 10 germinoma patients underwent both MET- and FDG-PET, and 2 only underwent FDG-PET. The patients’ age at diagnosis, sex, clinical presentation, and serum and CSF β-HCG and AFP levels; the duration of their symptoms; and information about the locations of the tumors and the administered treatments are summarized in Table 1.

**Table 1:** Patients characteristics and PET findings

						FDG				MET									
No	Age	Sex	Symptom	Location	Diagnosis	V.A.	SUV_max_	*T*/*N* (WM)	*T*/*N* (GM)	V.A.	SUV_max_	*T*/*N* (WM)	*T*/*N* (GM)	HCGs	AFPs	HCGc	AFPc	Treatment	Follow-up (month)
1	9	M	Left hemiparesis	Right basal ganglia	Clinical	3	4.3	1.9	0.8	2	2	3.3	1.4	9.6	(–)	(–)	(–)	CBDCA + VP-16 (3 course) Whole brain (24 Gy), ventricle (6 Gy) irradiation 30 Gy	54
2	12	M	Vomit, poor appetite	Left basal ganglia	Biopsy	3	4.6	1.8	0.8	3	0.6	2.8	2.4	(–)	(–)	(–)	(–)	CBDCA + VP-16 (3 course) whole brain irradiation 30 Gy	48
3	8	M	Left hemiparesis	Right basal ganglia	Clinical	4	6.8	1.7	1.1	3	2.2	2.4	1.8	4.6	(–)	19.4	(–)	CBDCA + VP-16 (3 course) whole brain irradiation 30 Gy	20
4	7	M	Left hemiparesis	Right basal ganglia	Biopsy	3	5.2	1.5	0.6	3	2.7	2.4	2.1	(–)	(–)	1.8	(–)	CBDCA + VP-16 (3 course) whole brain irradiation 30 Gy	48
5	10	F	Headache	Pineal	Biopsy	3	6.7	1.2	0.8	NA	NA	NA	NA	(–)	(–)	(–)	(–)	CBDCA + VP-16 (3 course) whole ventricle irradiation 30 Gy	42
6	13	F	Headache, DI	Suprasellar	Clinical	4	2.8	1.1	0.5	3	5.2	5.7	3.7	(–)	(–)	(–)	(–)	CBDCA + VP-16 (3 course) whole ventricle irradiation 30 Gy	24
7	29	M	Poor appetite, DI	Suprasellar	Clinical	2	2.2	0.7	0.4	1	0.9	1.2	0.9	(–)	(–)	3.5	(–)	CBDCA + VP-16 (3 course) whole ventricle irradiation 30 Gy	24
8	19	M	Vomit	Suprasellar, pineal	Clinical	4 (P, S)	6.1 (S) 6.9 (P)	2.2 (S) 2.5 (P)	0.85 (S) 0.96 (P)	NA	NA	NA	NA	(–)	(–)	(–)	(–)	CBDCA + VP-16 (3 course) whole ventricle irradiation 30 Gy	66
9	20	M	Headache, visual disorder	Suprasellar, pineal	Biopsy	3 (P)	9.8 (S)	1.6 (S)	0.95 (S)	3 (S,P)	1.9 (S)	1.7 (S)	1.2 (S)	(–)	(–)	(–)	(–)	CBDCA + VP-16 (3 course) Whole brain irradiation 30 Gy	60
						4 (S)	9.4 (P)	1.6 (P)	0.9 4(P)		2.1 (P)	1.9 (P)	1.3 (P)	(–)	(–)	(–)	(–)		
10	7	M	Headache, left hemiparesis	Left basal ganglia, pineal	Biopsy	2 (P)	5.1 (P)	1.6 (P)	0.6 4(P)	3 (P,B)	1.2 (P)	1.3 (P)	0.9 (P)	2.8	(–)	3.7	(–)	CBDCA + VP-16 (3 course) Whole brain irradiation 30 Gy	30
						4 (B)	5.9 (B)	1.8 (B)	0.7 4 (B)		2.0 (B)	2.2 (B)	1.5 (B)	(–)	(–)	(–)	(–)		

*DI* diabetes insipidus, *CBDCA* *+* *VP-16* carboplatin + etoposide, *s* suprasellar, *p* pineal, *b* basal ganglia, *V.A*. visual assessment, *SUV* standard uptake value, *T/N* ratio of tumor/normal, *GM* gray matter, *WM* white matter, *HCG* human chorionic gonadotropin, *s* serum, *c* cerebrospinal fluid, *AFP* alpha-fetoprotein, *s* serum, *c* cerebrospinal fluid, *NA* not available

One patient exhibited positivity for β-HCG in their serum alone (No. 1), two patients displayed positivity for β-HCG in their CSF alone (No. 4 and 7), and two patients showed positivity for β-HCG in both their serum and CSF (No. 3 and 10). Two out of 5 patients with elevated β-HCG levels were pathologically diagnosed with pure germinoma (No. 4 and 10). None of the patients displayed positivity for AFP in their serum or CSF.

We followed-up all of the patients (range 24–66 months; mean 41.6 months), and none of them suffered recurrence (as of February 2013).

### MRI results

All of the suprasellar and pineal tumors could be observed on Gd-enhanced T2-weighted images or T1-weighted MRI images. However, the tumors in the basal ganglia were not easy to detect or delineate because they had unclear boundaries and only produced slight signal changes. Therefore, it was difficult to measure the diameters of the basal ganglia germinomas. As for the suprasellar and pineal germinomas, their mean major diameter was 19 ± 7.0 (9–33) mm. The major diameter of tumor No. 7 was <10 mm, whereas those of the other tumors were more than 10 mm.

### PET results

**Fig. 1:**
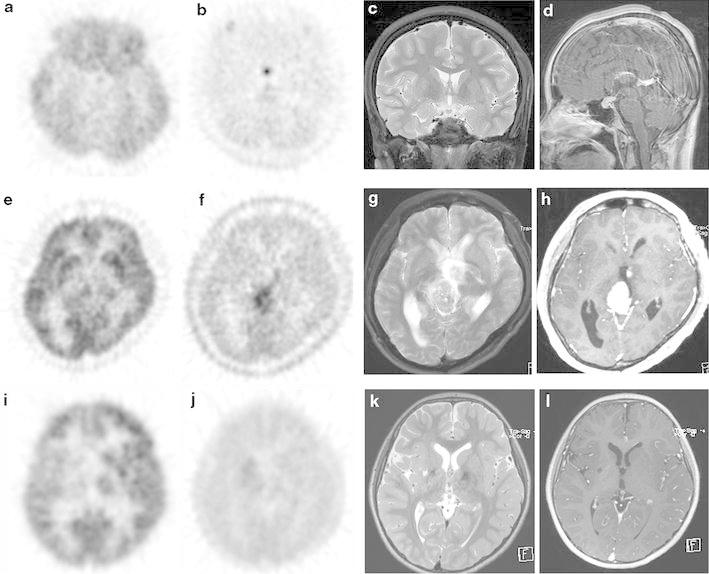
Representative case of CNS germinoma in the suprasellar region (No. 6). **a** FDG-PET detected slight FDG uptake. **b** MET-PET detected strong MET uptake. **c**, **d** T2-weighted images showing a small mass in the suprasellar region, and sagittal gadolinium-enhanced T1-weighted images showing subtle contrast enhancement. Representative case of a CNS germinoma in the pineal region (No. 9). **e** The tumor demonstrated an inhomogeneous uptake pattern, and the region displaying the strongest FDG-PET accumulation exhibited a similar uptake level to the gray matter. **f** MET-PET detected strong MET uptake. **g**, **h** T2-weighted images showing a mass in the pineal gland that exhibited inhomogeneous intensity on T2-weighted images, and gadolinium-enhanced T1-weighted images displaying strong enhancement. A suprasellar mass was simultaneously found in this case. Representative case of a CNS germinoma in the basal ganglia region (No. 4). **i** FDG-PET detected reduced FDG uptake in the right basal ganglia compared with the contralateral basal ganglia, and the FDG uptake of the tumor was intermediate between those of the normal white and gray matter. **j** The tumor demonstrated slight MET uptake on MET-PET. **k** T2-weighted images showing a region of increased signal intensity in the right basal ganglia, partial cystic changes, and right cerebral hemiatrophy. **l** On gadolinium-enhanced T1-weighted images, the tumor displayed very slight enhancement

The visual score, SUV_max_, *T*/*C* (WM), and *T*/*C* (GM) values for each case according to MET-PET and FDG-PET are summarized in Table 1. Representative images are shown in Fig. 1.

### MET-PET

According to MET-PET, the mean and SD values of the visual score, SUV_max_, *T*/*C* (WM), and *T*/*C* (GM) for all patients were 2.7 ± 0.7, 1.9 ± 1.4, 2.5 ± 1.3, and 1.7 ± 0.9, respectively (Table 2). On the other hand, the mean values and SD values of the visual score, SUV_max_, *T*/*C* (WM), and *T*/*C* (GM) for the patients who were pathologically diagnosed with pure germinoma were 3.0 ± 0, 1.8 ± 0.7, 2.1 ± 0.5, and 1.6 ± 0.6, respectively. There were no significant differences in any parameter between the values for all patients and those for the patients who were pathologically diagnosed with pure germinoma. The tumors were generally well delineated by MET-PET, except in one patient (No. 7), who had a germinoma in the suprasellar region. As for the tumors in the basal ganglia, all of them presented with similar MET uptake levels. On the other hand, the MET uptake values of the suprasellar and pineal tumors displayed inter-lesional differences. In almost all cases, the tumor exhibited stronger MET tracer uptake than the normal white matter and gray matter tissue.

**Table 2:** The uptake of PET at each region

	Total (12)	Suprasellar region (4)	Pineal region (4)	Basal ganglia	Pathological verified region (7)
FDG					
V.A.	3.3 ± 0.8	3.5 ± 1.0	3.0 ± 0.8	3.4 ± 0.5	3.1 ± 0.7
SUV_max_	5.8 ± 2.2	5.2 ± 3.4	7.0 ± 1.8	5.4 ± 1.0	6.7 ± 2.1
*T*/*N* (WM)	1.6 ± 0.5	1.4 ± 0.6	1.7 ± 0.6	1.7 ± 0.2	1.6 ± 0.2
*T*/*N* (GM)	0.8 ± 0.2	0.7 ± 0.3	0.8 ± 0.1	0.8 ± 0.2	0.8 ± 0.1
MET					
V.A.	2.7 ± 0.7	2.3 ± 1.2	3.0 ± 0.0	2.8 ± 0.4	3.0 ± 0
SUV_max_	1.9 ± 1.4	2.7 ± 2.3	1.7 ± 0.6	1.5 ± 1.2	1.8 ± 0.7
*T*/*N* (WM)	2.5 ± 1.3	2.9 ± 2.5	1.6 ± 0.4	2.6 ± 0.4	2.1 ± 0.5
*T*/*N* (GM)	1.7 ± 0.9	1.9 ± 1.5	1.1 ± 0.3	1.8 ± 0.4	1.6 ± 0.6

Uptake of FDG-PET and MET-PET in suprasellar, pineal and basal ganglia regions. Uptake was evaluated by visual assessment (V.A.) SUV_max_, *T*/*N* (WM), and *T*/*N* (GM)

*SUV* standard uptake value, *T/N* ratio of tumor/normal, *GM* gray matter, *WM* white matter

### FDG-PET

According to FDG-PET, the mean and SD values of the visual score, SUV_max_, *T*/*C* (WM), and *T*/*C* (GM) for all patients were 3.3 ± 0.8, 5.8 ± 2.2, 1.6 ± 0.5, and 0.8 ± 0.2, respectively (Table 2). On the other hand, the mean and SD values of the visual score, SUV_max_, *T*/*C* (WM), and *T*/*C* (GM) for the patients who were pathologically diagnosed with pure germinoma were 3.1 ± 0.7, 6.7 ± 2.1, 1.6 ± 0.2, and 0.8 ± 0.1, respectively. There was no significant difference in any parameter between the values for all patients and those for the patients who were pathologically diagnosed with pure germinoma. Similarly to MET-PET, the inter-lesion differences in SUV_max_ were small in the basal ganglia; however, the SUV_max_ values for the suprasellar and pineal tumors varied. In almost all cases, the tracer uptake of the tumor was intermediate between those of the normal white matter and normal gray matter.

## Discussion

Germinomas do not necessarily have to be diagnosed pathologically. In fact, they are often diagnosed based on the patient’s background, the results of radiological imaging or cerebrospinal fluid tests, and/or the patient’s serum tumor marker levels. In addition, because of their sensitivity to chemotherapy a small dose of chemotherapy is often administered for diagnostic and therapeutic purposes. Accordingly, the present study was conducted to examine whether PET could be used as an additional diagnostic tool for germinoma.

There are few case reports about the FDG-PET findings of CNS germinomas. Sadamura et al. and Yu et al. reported a case of CNS germinoma that was diagnosed using FDG-PET [23, 24]. In addition, there have been some reports about the utility of MET-PET for diagnosing CNS germinoma; however, these reports were restricted to tumors involving the basal ganglia [9, 11, 16]; therefore, little is known about the MET-PET and FDG-PET findings of germinoma and the effectiveness of these modalities for diagnosing germinoma in clinical practice.

Recently, Lee et al. [15] reported that MET-PET was useful for assessing the treatment responses of 3 basal ganglia germinomas Kawai et al. [11, 12] also reported that MET-PET is useful for response assessment and biopsy planning. Lee et al. assessed tracer uptake using the T/N ratio and SUV_max_, and Kawai et al. assessed it using SUV_max_. In these reports, SUV_max_ values of 1.5–2.5 were observed in pretreatment examinations, and the *T*/*N* ratio ranged from 1.5 to 2.5, which were similar to our results.

In our study, all of the lesions except one were positively identified by MET-PET, suggesting that MET is a good tracer for detecting CNS germinoma. In particular, MET-PET is considered to be better than CT and MRI at detecting lesions in the basal ganglia, as such lesions only exhibit subtle signal changes or no mass effects on CT and MR images. In the present study, the lesion that produced a negative result on MET-PET measured less than 10 mm in diameter (the smallest tumor in the current study).

Furthermore, the germinoma lesions examined in the present study exhibited markedly higher MET uptake compared with the normal brain tissue, which enabled us to obtain a high level of contrast during tumor imaging. In the clinical setting, MET-PET is considered to be useful for tumor contouring and treatment planning.

For example, it is possible to conduct more accurate biopsies based on MET-PET, especially in the basal ganglia region. Radiation therapy is the standard treatment for germinoma, and the treatment field is chosen based on the contours of the tumor. Therefore, it is very important to accurately determine the location and borders of the tumor. MET-PET is considered to be a good imaging tool for detecting germinoma.

Further studies should be conducted on other uses for MET-PET imaging, for example, its utility for differential diagnosis, tumor grading, or recurrence diagnosis, etc., in germinoma.

To the best of our knowledge, only case reports have described the use of FDG-PET to diagnose CNS germinoma, and there have not been any systematic studies of this issue; therefore, the tracer uptake values of CNS germinomas on FDG-PET are unknown. In the present study, the CNS germinomas generally showed uptake values that were intermediate between those of the gray and white matter. In addition, the visual appearance of the CNS germinomas differed depending on their location, even at similar uptake levels. Namely, the tumors in the suprasellar and pineal regions usually exhibited greater tracer uptake than the surrounding brain tissue or cerebrospinal fluid; on the other hand, those in the basal ganglia exhibited reduced tracer uptake compared with the surrounding normal basal ganglia in the normal hemisphere. Thus, we must be careful when evaluating FDG uptake visually.

In 1999, Utsuki et al. [25] reported the relationship between the serum level of β-HCG and the prognoses of various germinomas, including pure germinoma and germinoma with STGC. In the latter report, germinoma patients with serum β-HCG levels of <15 mIU/ml exhibited a high recurrence rate; however, the remaining patients did not suffer recurrence and achieved good outcomes. In 2002, the same group suggested that increased CSF β-HCG levels, but not increased serum β-HCG levels, are a favorable prognostic factor in cases of germinoma and that germinoma patients who exhibit such findings have similar recurrence rates and prognoses to those with pure germinomas [26]. In our study, 5 of 10 patients were diagnosed based on their clinical features, rather than by biopsy; therefore, there is a possibility that some of these patients had germinoma with STGC. These patients demonstrated increased β-HCG levels in their CSF (No. 4 and 7) or serum (No. 1, 3, and 10); however, their serum β-HCG levels were much <15 mIU/ml. Therefore, according to previous reports it was expected that our patients would exhibit a good prognosis, even if some of the lesions were germinoma with STGC. In fact, in the present study there was no difference in the uptake of MET or FDG between the values for all germinoma patients and those for the patients that were diagnosed pathologically. Subsequently, all of the tumors exhibited good responses to therapy, and none of the patients suffered recurrence during the observation period.

There were some limitations to this study. First, the sample size was small, and the study had a retrospective design. In addition, there were inter-individual variations in tumor size, and the tracer uptake of small lesions is not easy to measure accurately due to the partial volume effect. Second, it is possible that the tumors examined in the present study included some CNS germinomas with STGC.

## Conclusion

In the present study, we demonstrated the FDG-PET and MET-PET findings of CNS germinoma (possibly including those of germinoma with STGC). On MET-PET, almost all of lesions could be visualized; therefore, MET is considered to be a good tracer for diagnosing CNS germinoma, especially for tumor contouring. Although the CNS germinomas could be detected by FDG-PET, the image contrast was poor due to tracer uptake by the surrounding normal brain tissue. We consider that further studies are needed before FDG-PET can be used in clinical examinations of CNS germinoma.
